# The TGF‐β‐induced up‐regulation of NKG2DLs requires AKT/GSK‐3β‐mediated stabilization of SP1

**DOI:** 10.1111/jcmm.13025

**Published:** 2017-02-06

**Authors:** Xiao‐Hui Chen, Lin‐lin Lu, Hong‐Peng Ke, Zong‐Cai Liu, Hai‐Fang Wang, Wei Wei, Yi‐Fei Qi, Hong‐Sheng Wang, Shao‐Hui Cai, Jun Du

**Affiliations:** ^1^ Department of Microbial and Biochemical PharmacySchool of Pharmaceutical SciencesSun Yat‐sen UniversityGuangzhouChina; ^2^ Department of PharmacologySchool of Pharmaceutical SciencesJinan UniversityGuangzhouChina

**Keywords:** NK cells, SP1, NK group 2 member D, transforming growth factor beta, cancer

## Abstract

Natural killer (NK) cells play an important role in preventing cancer development. NK group 2 member D (NKG2D) is an activating receptor expressed in the membrane of NK cells. Tumour cells expressing NKG2DL become susceptible to an immune‐dependent rejection mainly mediated by NK cells. The paradoxical roles of transforming growth factor beta (TGF‐β) in regulation of NKG2DL are presented in many studies, but the mechanism is unclear. In this study, we showed that TGF‐β up‐regulated the expression of NKG2DLs in both PC3 and HepG2 cells. The up‐regulation of NKG2DLs was characterized by increasing the expression of UL16‐binding proteins (ULBPs) 1 and 2. TGF‐β treatment also increased the expression of transcription factor SP1. Knockdown of SP1 significantly attenuated TGF‐β‐induced up‐regulation of NKG2DLs in PC3 and HepG2 cells, suggesting that SP1 plays a key role in TGF‐β‐induced up‐regulation of NKG2DLs. TGF‐β treatment rapidly increased SP1 protein expression while not mRNA level. It might be due to that TGF‐β can elevate SP1 stability by activating PI3K/AKT signalling pathway, subsequently inhibiting GSK‐3β activity and decreasing the association between SP1 and GSK‐3β. Knockdown of GSK‐3β further verified our findings. Taken together, these results revealed that AKT/GSK‐3β‐mediated stabilization of SP1 is required for TGF‐β induced up‐regulation of NKG2DLs. Our study provided valuable evidence for exploring the tumour immune modulation function of TGF‐β.

## Introduction

Natural killer (NK) cells belong to type of lymphocytes that mediate innate immunity against pathogens and tumours 1. NK cells play an important role in preventing cancer development by eliminating suspicious cells 2. The function of NK cells is regulated by a balance between signals transmitted by cell‐surface inhibitory and activating receptors on NK cells 1, 3. The inhibitory receptors are specific for major histocompatibility complex class I molecules^4^. The activating receptors, such as NK group 2 member D (NKG2D), recognize ligand on tumours and virus‐infected cells 1, 3, 4, 5. NKG2D is an activating receptor expressed by both NK cells and cytolytic T lymphocyte (CTL) cells, which plays a critical role in elimination of tumour cells 6, 7. In NK cells, NKG2D serves as a primary activation receptor, which is able to trigger cytotoxicity. In CTL cells, NKG2D acts as a costimulatory receptor of TCR‐activated cells.

In human, the ligands of NKG2D (NKG2DLs) are MHC class I chain‐related molecules A and B (MICA/B) and UL16‐binding protein (ULBP) molecules, whose expression are restricted in normal tissues while abnormally increased in cancer and infected cells 8, 9, 10. Cancer cells expressing NKG2DLs become susceptible to the cytotoxic lysis mediated by NK and T cells 11. As to the expression of MICA/B and ULBP1 in tumour cell lines, it is reported that transcription factors SP1, SP3, NF‐Y and the stress‐inducible heat‐shock factor 1 (HSF1) can activate their transcription 12, 13, 14. For example, SP1 transcription factor is a crucial regulator of NKG2DL transcription through its binding to GC boxes located in their promoters. Although NKG2DLs can be elevated by DNA damage, heat shock and oxidative stress 15, 16, 17, the detailed mechanisms about the regulation of NKG2DLs are not well understood.

Transforming growth factor beta (TGF‐β) plays a pleiotropic role in the tumour progression, from a tumour suppressor in less advanced tumours and healthy cells to a promoter in more aggressive cancers 18. Elevated level of TGF‐β in microenvironment is related to the immune system. TGF‐β is recognized as one of the most potent immunosuppressive agents in facilitating oncogenesis 19. The paradoxical roles of TGF‐β in regulation of NKG2DL are presented in many studies 20, 21. Some studies said that TGF‐β can suppress the expression of NKG2DL 20, while others reported that TGF‐β can up‐regulate the expression of NKG2D 21. In addition, the detailed mechanisms are unclear. Here, we showed that TGF‐β induced the up‐regulation of NKG2DLs by stabilizing SP1 in PC3 and HepG2 cells. We also demonstrated that TGF‐β can up‐regulate SP1 *via* TGF‐β receptor through activating PI3K/AKT signalling pathway, subsequently inhibiting GSK‐3β activity and decreasing the association of SP1 with GSK‐3β.

## Materials and methods

### Chemicals and reagents

Recombinant human TGF‐β protein was obtained from PeproTech (Rocky Hill, NJ, USA). Primary antibodies against GSK‐3β (27C10), GSK‐3β (Ser9), AKT (C67E7), p‐AKT (Ser473) were purchased from Cell Signaling Technology (Danvers, MA, USA). Primary antibody against SP1(G447) was obtained from Bioworld (Bioworld Technology, Minneapolis, MN, USA). Protein A/G sepharose and primary antibodies against ubiquitin (SC‐166553) and β‐actin (SC‐130300) were obtained from Santa Cruz Biotechnology (Santa Cruz, CA, USA). Phycoerythrin (PE)‐labelled antibody against ULBP1 and ULBP2 were obtained from R&D (San Diego, CA, USA). Horseradish peroxidase (HRP)‐conjugated secondary antibody, Alexa Fluor 488‐conjugated secondary antibody, 4′, 6‐diamidino‐2‐phenylindole (DAPI) and lipofectamine 2000 were purchased from Invitrogen (Carlsbad, CA, USA). Proteasome inhibitor MG132 was obtained from Sigma‐Aldrich (St Louis, MO, USA). PrimeScript RT reagent Kit and SYBR Premix Ex TaqTM were products of TaKaRa. E.Z.N.A HP Total RNA Kit was purchased from Omega Bio‐Tek (Doraville, GE, USA). Smart pool siRNA against human SP1 (siSP1), GSK‐3β (si‐GSK‐3β) and control (siNC) were purchased from RiboBio (Guangzhou, China). ERK inhibitor PD98059, p38 MAPK inhibitor SB203580, PI3K inhibitor LY294002 and TGF‐β type I receptor inhibitor SB431542 were obtained from Sigma‐Aldrich.

### Cell culture

The human prostate cancer PC3 and hepatocellular carcinoma HepG2 cells were obtained from the Type Culture Collection of the Chinese Academy of Sciences (Shanghai, China). Cells were maintained in RPMI 1640 supplemented with 10% fetal bovine serum, 100 μg/ml streptomycin and 100 units/ml penicillin under a humidified 5% CO_2_ atmosphere at 37°C in an incubator.

### Flow cytometry

After different treatments, cells were harvested by trypsinization using 0.05% trypsin–EDTA and washed twice with PBS. Then, cells were stained for 30 min. at 4°C and dark with optimal dilution of PE‐conjugated mAb or isotope control mAb. Then cells were washed, resuspended with phosphate‐buffered saline (PBS) and analysed using flow cytometry (EXL^TM^; Beckman Coulter, Chaska, MN, USA).

### Quantitative real‐time PCR

Quantitative real‐time PCR was performed as previously described 22. Total mRNA of the cells was extracted after treatment for the indicated times. Briefly, total mRNA of the cells was extracted after treatment for the indicated times. First‐strand cDNA synthesis was generated from 500 ng of total RNA. Quantification of target and reference (GAPDH) genes was performed in triplicate on LightCyclerH 480 II (Roche, Applied Science, Basel, Switzerland). The primers used in each reaction were as follows: SP1 forward 5′‐AGA ATC AGG AGA GGG ATA CAA GAG A‐3′and reverse 5′‐GTA AGA AGA AAC TGG GGT CAA ACA A‐3′; MICA/B forward 5′‐ACA TGG AAT GTC TGC CAA TGA CT ‐3′ and reverse 5′‐AAT GGA ACC TAC CAG ACC TGG G ‐3′; ULBP1, forward 5′‐ATC AGC GCC TCC TGT CCA C‐3′ and reverse 5′‐AAA GAC AGT GTG TGT CGA CCC AT‐3′; ULBP2, forward 5′‐CCT AGC GCT CTG GGT CCT T‐3′ and reverse 5′‐AAA GAG AGT GAG GGT CGG CTC‐3′; ULBP3, forward 5′‐GCC TCG CGA TTC TTC CGT A‐3′ and reverse 5′‐GTG ACC CAT AGA TAA GAC CTT GTC AC’; GAPDH, forward 5′‐GCA CCG TCA AGG CTG AG AAC‐3′ and reverse 5′‐TGG TGA AGA CGC CAG TGG A‐3′. After normalized to GAPDH gene, expression levels for each target gene were calculated using the comparative threshold cycle (CT) method. The Δct values were calculated according to the formula Δct = ct (gene of interest)‐ct (GAPDH) in correlation analysis, and the 2‐ΔΔct was calculated according to the formula ΔΔct = Δct (control group)−Δct (experimental group) for determination of relative levels.

### Western blot analysis

Western blot analysis was performed as previously described 23. Briefly, cells were washed three times with ice‐cold PBS and then lysed in lysis buffer containing 50 mM Tris–HCl (pH 7.6), 150 mM NaCl, 1 mM EDTA, 1% NP‐40, 0.5% Na‐deoxycholate, 5 μg/ml aprotinin, 5 μg/ml leupeptin and 1 mM phenylmethylsulfonyl fluoride. Lysates were cleared by centrifugation and denatured by boiling in Laemmli buffer. Equal amounts of protein samples were loaded per well and separated on SDS–polyacrylamide gels, and then electrophoretically transferred onto polyvinylidene fluoride (PVDF) membranes. Following blocking with 5% non‐fat milk at room temperature for 2 hrs, membranes were incubated with primary antibodies (1:1000 dilution) at 4°C overnight and then incubated with horseradish peroxidase (HRP)‐conjugated secondary antibodies (1:5000 dilution) for 2 hrs at room temperature. Specific immune complexes were detected using Western Blotting Plus Chemiluminescence Reagent (Life Science, St Petersburg).

### Immunoprecipitation

Immunoprecipitation was performed as previously described 24. Briefly, cells were washed three times with ice‐cold PBS and harvested at 4°C in immunoprecipitation lysis buffer containing 50 mM HEPES, pH 7.5, 150 mM NaCl, 0.5% NP‐40, 2 mM EDTA, 10% glycerol, 1 mM Na_3_VO_4_, 1 mM NaF, 1 mM dithiothreitol, 1 mM 4‐(2‐aminoethyl) benzenesulfonyl fluoride, 1 μg/ml leupeptin, 1 μg/ml pepstatin and 1 μg/ml aprotinin. Equal amounts of protein were immunoprecipitated using anti‐SP1 antibody, and the immune complexes were bound to protein A/G sepharose. The beads were washed with lysis buffer and subjected to Western blotting with anti‐ubiquitin or anti‐GSK‐3β antibodies.

### Statistical analyses

Results were expressed as mean ± standard deviation (S.D.) of three independent experiments unless otherwise specified. Data were analysed by nonparametric test two‐tailed Wilcoxon rank‐sum test between two groups. These analyses were performed with GraphPad Prism Software version 5.0 (GraphPad Software Inc., La Jolla, CA, USA). Immunofluorescence results were analysed by Image‐Pro Plus 6.0. FlowJo software A (Ashland, OR, USA) was used to analyse the data of flow cytometry. *P*‐value of <0.05 was considered as statistically significant.

## Results

### TGF‐β up‐regulates the expression of NKG2DLs in both PC3 and HepG2 cells

**Figure 1:**
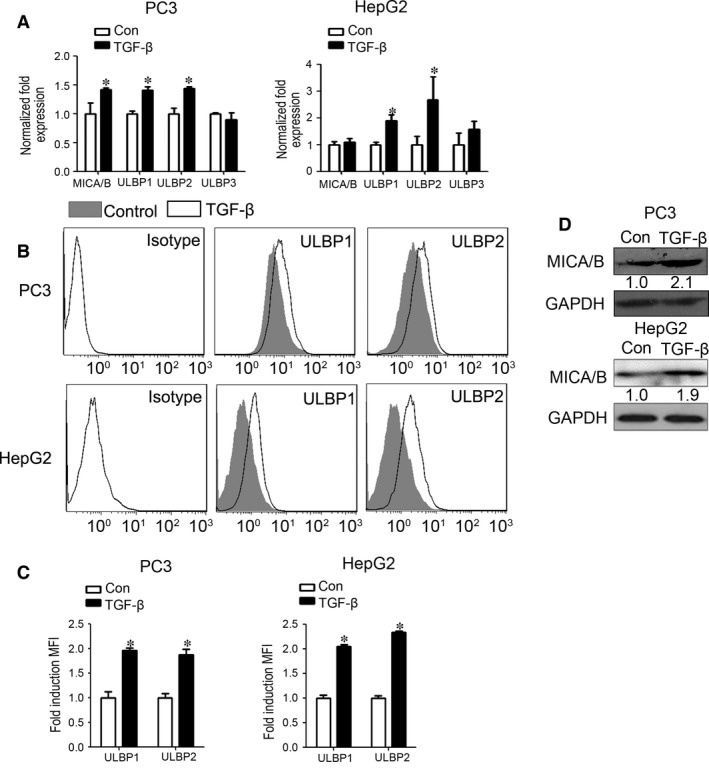
Transforming growth factor beta (TGF‐β) up‐regulates the expression of NKG2DLs in both PC3 and HepG2 cells. (**A**) PC3 and HepG2 cells were treated with or without TGF‐β (10 ng/ml) for 24 hrs, and then the mRNA levels of MICA/B, ULBP1, ULBP2 and ULBP3 were analysed by qRT‐PCR. (**B**) PC3 and HepG2 cells were treated with or without TGF‐β (10 ng/ml) for 3 days, and then the expression of ULBP1 and ULBP2 was determined by flow cytometry. (**C**) And fold induction (MFI) was calculated to show the ULBP1 and ULBP2 expression differences between groups. (**D**) PC3 and HepG2 cells were treated with or without TGF‐β (10 ng/ml) for 3 days, and then the expression of MICA/B was determined by Western blotting. Data represent the average of three independent experiments. *P < 0.05 compared with control.

To study the effects of TGF‐β on the expression of NKG2DLs in cancer PC3 and HepG2 cells, the mRNA expression and protein expression of NKG2DLs were evaluated. The results showed that the mRNA expression of ULBP1 and ULBP2 was significantly enhanced in PC3 and HepG2 cells after TGF‐β treatment for 24 hrs (Fig. 1A). Flow cytometry results confirmed that protein levels of MICA/B and ULBP1, ULBP2 were significantly up‐regulated in PC3 and HepG2 cells (Fig. 1B and C). Western blotting result also showed that the protein expression of MICA/B level was up‐regulated (Fig. 1D). The results indicated that TGF‐β is able to induce the expression of NKG2DLs in cancer cells.

### SP1 is crucial for TGF‐β induced up‐regulation of NKG2DLs

**Figure 2:**
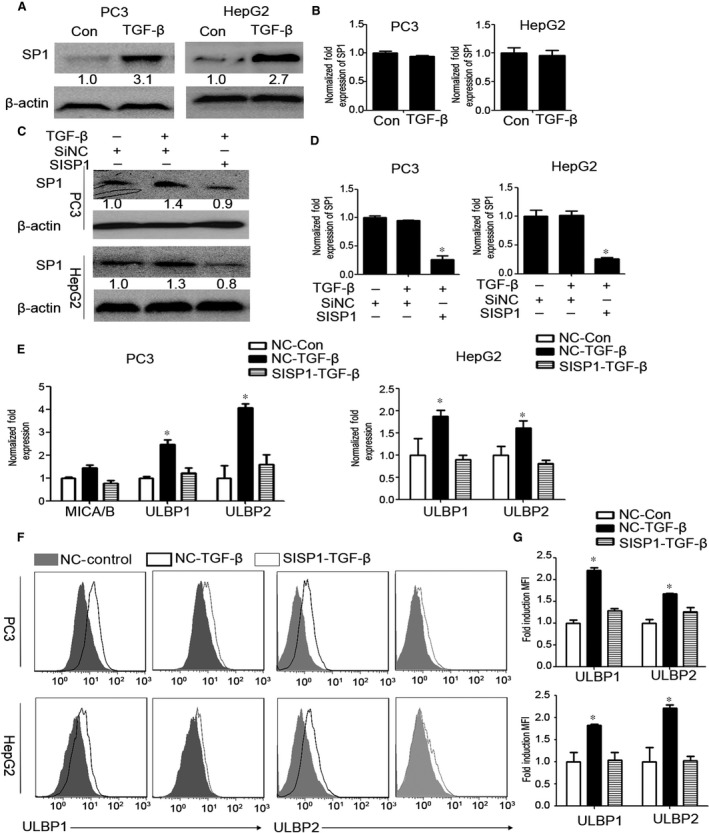
SP1 is crucial for transforming growth factor beta (TGF‐β)‐induced up‐regulation of NKG2DLs. (**A**) PC3 and HepG2 cells were treated with or without TGF‐β (10 ng/ml) for 24 hrs, and then the protein expression of SP1 was analysed by Western blotting. β‐actin servers as the loading control. (**B**) PC3 and HepG2 cells were treated with or without TGF‐β (10 ng/ml) for 24 hrs, and the mRNA levels of SP1 were analysed by qRT‐PCR. (**C**) After 24 hrs pre‐transfection with Si‐SP1 or Si‐NC siRNAs, PC3 and HepG2 cells were further stimulated with or without TGF‐β (10 ng/ml) for 24 hrs, and then the expression of SP1 was analysed by Western blotting. β‐actin servers as the loading control. (**D**) After 24 hrs pre‐transfection with Si‐SP1 or Si‐NC siRNAs, PC3 and HepG2 cells were further stimulated with or without TGF‐β (10 ng/ml) indicated for 24 hrs, and then the expression of SP1 at mRNA level was analysed by qRT‐PCR. (**E**) After 24 hrs pre‐transfection with Si‐SP1 or Si‐NC siRNAs, PC3 and HepG2 cells were further stimulated with or without TGF‐β (10 ng/ml) indicated for 24 hrs, and then the mRNA levels of ULBP1 and ULBP2 were analysed by qRT‐PCR. (**F**) After 24 hrs pre‐transfection with Si‐SP1 or Si‐NC siRNAs, PC3 and HepG2 cells were further stimulated with or without TGF‐β (10 ng/ml) for 3 days, and then the expression of ULBP1 and ULBP2 was determined by flow cytometry. (**G**) And fold induction (MFI) was calculated to show the ULBP1 and ULBP2 expression differences between groups. Data represent the average of three independent experiments. *P < 0.05 compared with control.

As previous studies have been shown that transcription factor SP1 is a crucial regulator of NKG2DL transcription through its binding to GC boxes located in their promoters 12, 13, 14, 25, we then investigated whether expression of SP1 was up‐regulated in PC3 and HepG2 after treated with TGF‐β. Compared with untreated cells, TGF‐β significantly increased the protein, but not mRNA levels of SP1 (Fig. 2A and B). We further performed knockdown assays to verify the role of SP1 in TGF‐β induced expression of NKG2DLs. SP1 was successfully knocked down by RNA interfering in PC3 and HepG2 cells (Fig. 2C and D) and then treated with TGF‐β for different times. Compared with control, silencing of SP1 attenuated TGF‐β‐induced expression of NKG2DLs, while protein and mRNA levels of ULBP1 were totally restored, and the protein and mRNA of ULBP2 were partially restored in TGF‐β treated PC3 and HepG2 cells (Fig. 2E–G). Taken together, these observations indicated that SP1 is crucial for TGF‐β mediated up‐regulation of NKG2DLs in cancer cells.

### TGF‐β regulates the protein stability and cellular localization of SP1

**Figure 3:**
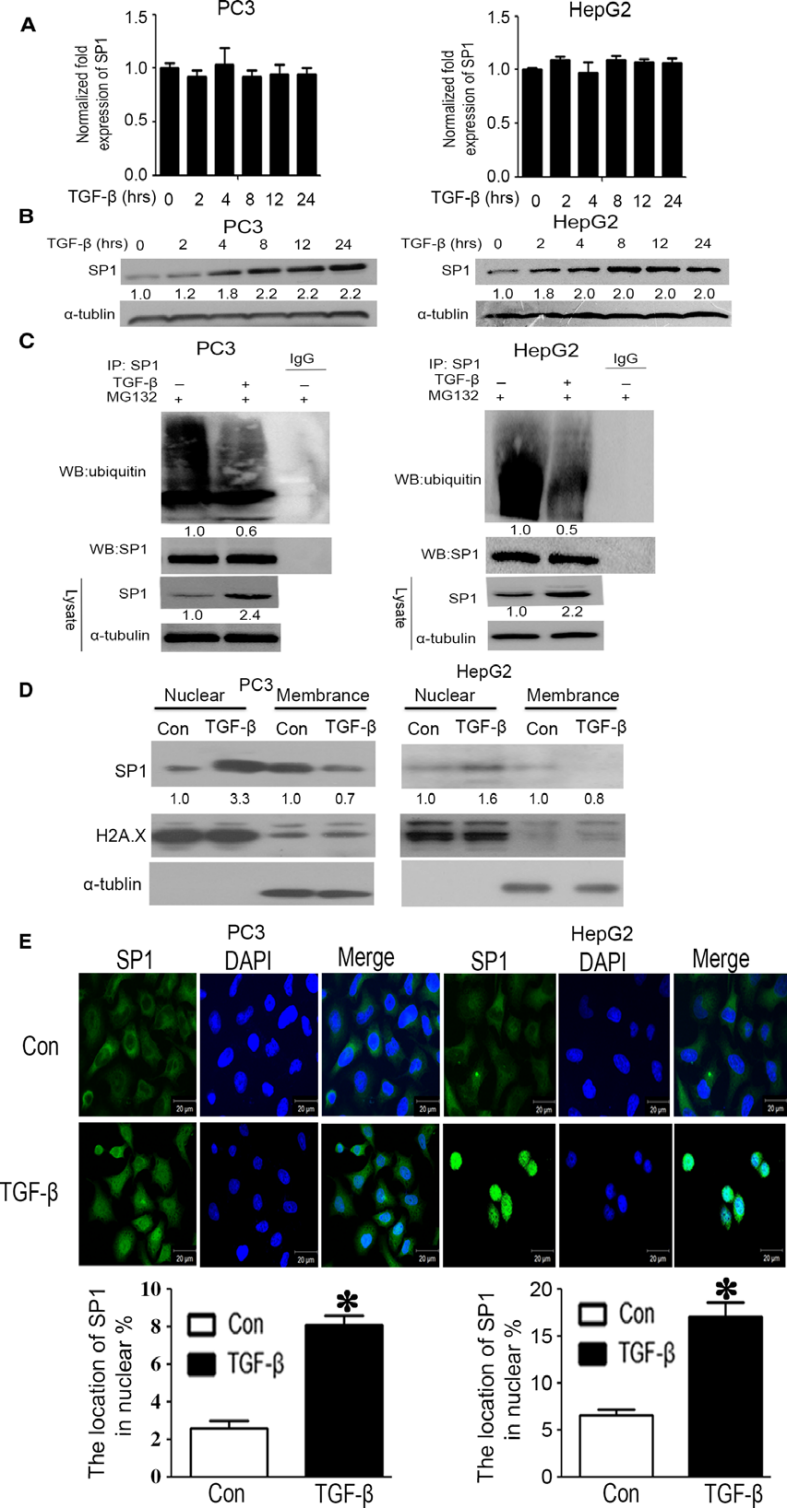
Transforming growth factor beta (TGF‐β) regulates the protein stability and cellular localization of SP1. (**A**) PC3 and HepG2 cells were treated with TGF‐β (10 ng/ml) for 0, 2, 4, 8, 12 and 24 hrs, and the mRNA levels of SP1 were examined by qRT‐PCR. (**B**) PC3 and HepG2 cells were treated with TGF‐β (10 ng/ml) for 0, 2, 4, 8, 12 and 24 hrs, and the protein levels of SP1 were examined by Western blotting. (**C**) PC3 and HepG2 cells were treated with TGF‐β (10 ng/ml) and MG132 (10 mM) for 8 hrs. After SP1 was immunoprecipitated from equal amount of lysates (two lower panels), the ubiquitination of SP1 was examined by Western blotting. (**D**) PC3 and HepG2 cells were treated with TGF‐β (10 ng/ml) for 24 hrs, SP1 located at cytoplasm and nucleus were isolated, respectively, and then analysed by Western blotting. (**E**) PC3 and HepG2 cells were treated with or without TGF‐β (10 ng/ml) for 8 hrs. After fixation, the cellular location of SP1 (green) was examined by immunofluorescence staining. Nuclei were stained with DAPI (blue). Scale bars: 20 mm. The results of immunofluorescence were analysed by image‐pro plus 6.0 software.

That TGF‐β increased SP1 protein but not mRNA suggested that up‐regulation of SP1 by TGF‐β was occurring at the post‐transcriptional level. To further verify this hypothesis, PC3 and HepG2 cells were treated with TGF‐β for 0–24 hrs, and then SP1 protein and mRNA levels were detected by flow cytometry and qRT‐PCR, respectively. The results showed that SP1 has no significant variation at mRNA level (Fig. 3A), but an obvious up‐regulation at protein level (Fig. 3B), which indicated that the up‐regulation of SP1 by TGF‐β might be due to protein stabilization. Because the protein stability of SP1 is regulated *via* ubiquitin‐mediated proteasomal degradation processes, we speculated whether the stabilization of SP1 by TGF‐β is mediated by suppression of SP1 ubiquitylation. The results indicated that TGF‐β suppressed the ubiquitylation of SP1 as compared with the control group, although total stabilized SP1 proteins were parallel (Fig. 3C). Furthermore, we found that the increased SP1 were almost located in nuclear instead of the cytoplasm (Fig. 3D). We also determined the nuclear translocation activity of SP1 by immunofluorescence. As shown in Fig.3E, compared with the control group, TGF‐β significantly increased the nuclear translocation of SP1. Taken together, our results revealed that TGF‐β can increase the expression of SP1 by suppression of SP1 ubiquitylation and then triggering its nuclear translocation.

### TGF‐β promotes expression of SP1 *via* PI3K/AKT signal pathway

**Figure 4:**
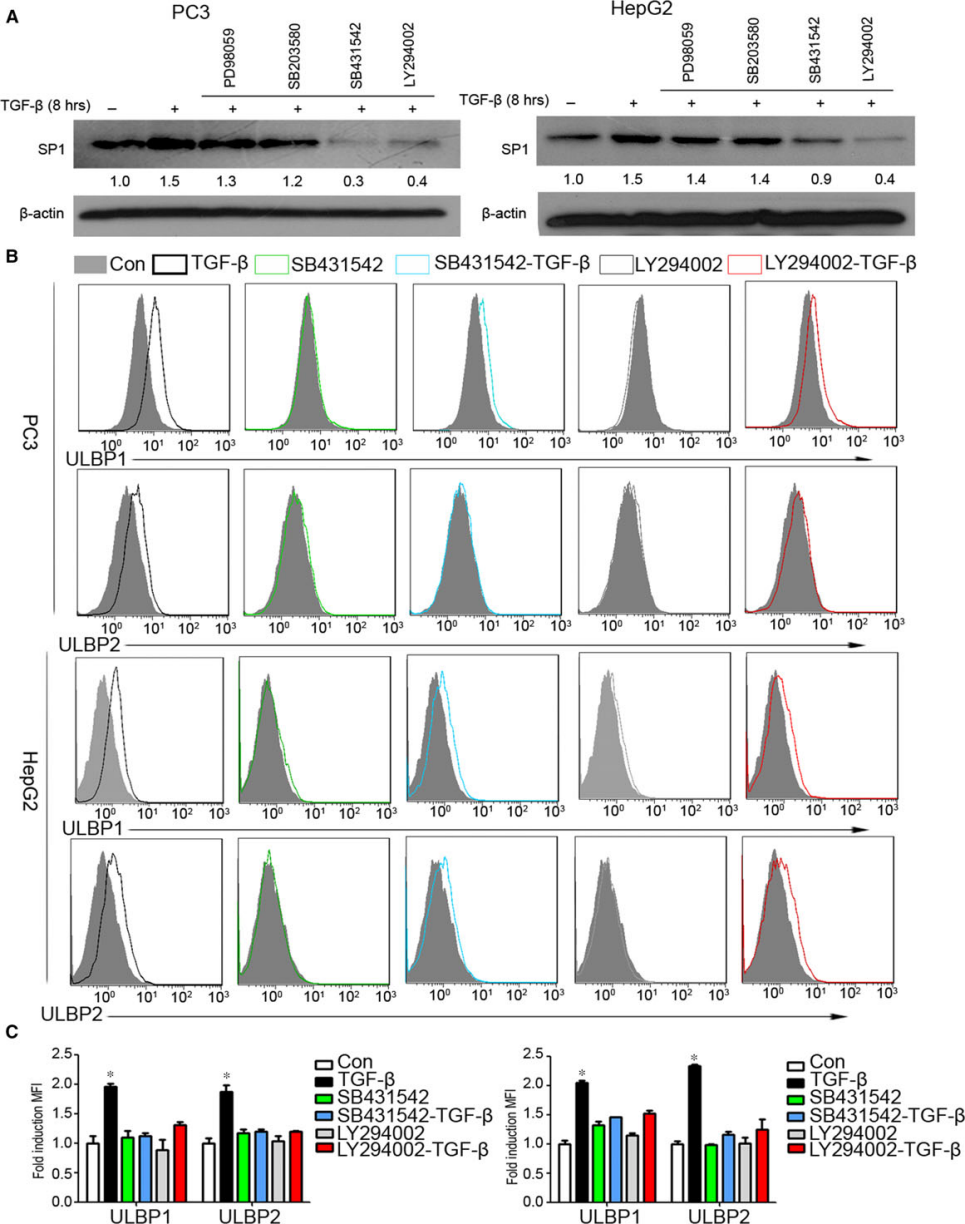
Transforming growth factor beta (TGF‐β) promotes expression of SP1 *via* PI3K/AKT signal pathway. (**A**) PC3 and HepG2 cells were pre‐treated with SB‐203580 (20 mM), PD98059 (20 mM), SB431542 (20 mM), LY294002 (20 mM) for 1 hr, respectively, followed by stimulation with TGF‐β (10 ng/ml) for 8 hrs. The expression of SP1 at protein level was examined by Western blotting. (**B**) After 1 hr pre‐treated with SB431542 (20 mM), LY294002 (20 mM), PC3 and HepG2 cells were further stimulated with or without TGF‐β (10 ng/ml) for 3 days. The expression of ULBP1 and ULBP2 was determined by flow cytometry. (**C**) And fold induction (MFI) was calculated to show the ULBP1 and ULBP2 expression differences between groups. Data represent the average of three independent experiments. *P < 0.05 compared with control.

To investigate the molecular mechanisms underlying TGF‐β‐mediated SP1 stabilization, inhibitors of PI3K/AKT (LY294002), MAPK (PD98059), p38 (SB‐203580) and TGF‐β type I receptor inhibitor (SB431542) were used to pre‐treatment of cells, as TGF‐β can induce the activation of these pathways and receptor. We found block TGF‐β type I receptor can partially restore the up‐regulation of SP1. More importantly, we found that PI3K/AKT inhibitor (LY294002), but not the others, completely blocked TGF‐β stabilized SP1 (Fig. 4A), suggesting that the activation of the PI3K/AKT pathway is responsible for TGF‐β‐mediated SP1 stabilization.

To determine whether the up‐regulation of NKG2DLs by TGF‐β is mediated by TGF‐β type I receptor and PI3K/AKT pathway, PC3 and HepG2 cells were pre‐treated with inhibitor of TGF‐β type I receptor inhibitor (SB431542) or PI3K/AKT (LY294002) for 1 hr before TGF‐β stimulation, and then protein level expression of ULBP1 and ULBP2 was determined by flow cytometry. As shown in Fig. 4B and C, compared with control, cells treated with SB431542 or LY294002 solely had no effect on expression of NKG2DLs. While in PC3 and HepG2 cells were pre‐treated with SB431542 or LY294002, TGF‐β‐induced protein expression of NKG2DLs were partially restored. Taken together, these observations indicated that PI3K/AKT pathway is responsible for TGF‐β‐mediated SP1 stabilization and further confirmed that SP1 is crucial for TGF‐β‐mediated up‐regulation of NKG2DLs in cancer cells.

### TGF‐β promotes SP1 stabilization by inhibiting the association between SP1 and GSK‐3β

**Figure 5:**
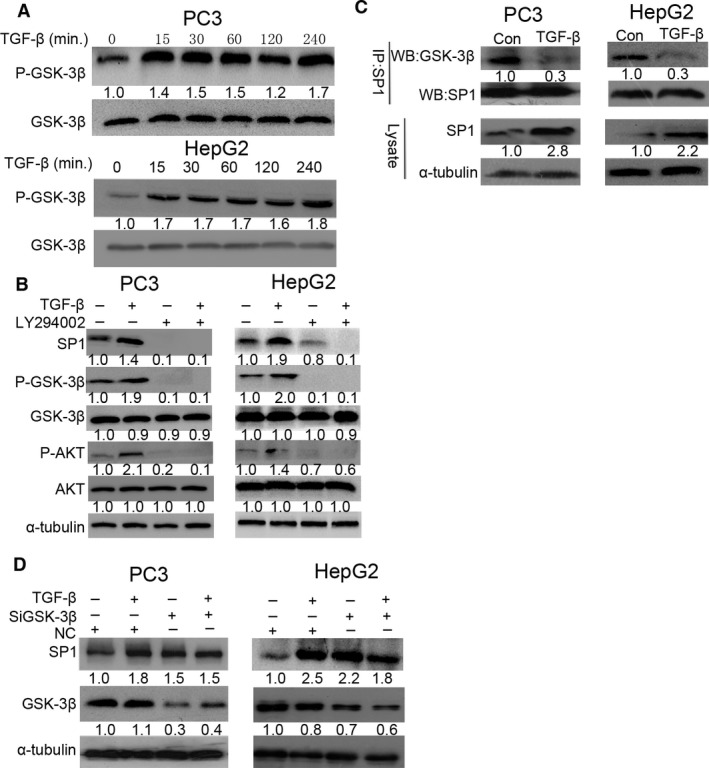
Transforming growth factor beta (TGF‐β) promotes SP1 stabilization by inhibiting the association of SP1 and GSK‐3β. (**A**) PC3 and HepG2 cells were treated with TGF‐β (10 ng/ml) for the times indicated. The expression of pGSK‐3β and GSK‐3β at protein level was examined by Western blotting. (**B**) PC3 and HepG2 cells were pre‐treated with or without LY294002 (20 mM) for 1 hr, followed by stimulation with or without TGF‐β (10 ng/ml) for 8 hrs. The expression of SP1 and the activation of AKT and GSK‐3β were examined by Western blotting. (**C**) PC3 and HepG2 cells were treated with TGF‐β (10 ng/ml) and MG132 (10 mM) for 8 hrs. After SP1 was immunoprecipitated from equal amount of lysates (two lower panels), the associated GSK‐3β was detected by Western blotting. (**D**) Cells were transfected with si‐NC or si‐GSK‐3β for 24 hrs, and then treated with or without TGF‐β (10 ng/ml) for additional 8 hrs. The expression of SP1 and GSK‐3β at protein level was examined by Western blotting.

As GSK‐3β is the main downstream kinase of AKT, we speculated whether the stabilization of SP1 by TGF‐β is associated with GSK‐3β, which maintains an active state in dephosphorylated form. We found that the expression of p‐GSK‐3β increased in a time‐dependent manner upon TGF‐β stimulation in PC3 and HepG2 cells (Fig. 5A). To determine whether the stabilization of SP1 by TGF‐β is mediated by regulation of GSK‐3β activity, we next treated PC3 and HepG2 cells with LY294002 prior to TGF‐β stimulation. We found that levels of p‐AKT, p‐ GSK‐3β and SP1 were increased after TGF‐β treatment for 8 hrs, while these effects were reversed upon treating with LY294002 alone or in combination with TGF‐β (Fig. 5B). We further studied the interaction between SP1 and GSK‐3β by immunoprecipitation. It showed that the binding of SP1 with GSK‐3β was markedly decreased in PC3 and HepG2 cells after stimulation with TGF‐β (Fig. 5C). These results suggested that the stabilization of SP1 by TGF‐β may be due to the inhibition of GSK‐3β activity. To further confirm our findings, we knocked the expression of GSK‐3β in PC3 and HepG2 cells using specific GSK‐3β siRNA (Fig. 5D). Compared with control, down‐regulation of GSK‐3β markedly elevated the protein levels of SP1. Taken together, these results demonstrated that TGF‐β up‐regulates SP1 in PC3 and HepG2 cells by activating AKT signalling and then phosphorylation of GSK‐3β.

## Discussion

The paradoxical roles of TGF‐β in regulation of NKG2DL and NKG2D are presented in many studies 20, 21. Some studies said that TGF‐β can suppress the expression of NKG2DL 20, while others reported that TGF‐β can up‐regulate the expression of NKG2D 21. In the immune system, tumour cells expressing NKG2DLs become susceptible to an immune‐dependent rejection mainly mediated by NK and T cells. NKG2D response may protect the host in the early stage of tumour development through activation of ataxia telangiectasia mutated pathway and up‐regulation of the expression of NKG2DL in tumour cells 26. Along with the development of tumour, advanced tumours and metastases develop several ways to evade NKG2D signalling including repression of NKG2DL transcription and shedding of soluble MHC class I chain‐related molecule A (sMICA) 16, 27. TGF‐β is a pleiotropic cytokine that exerts dual roles in tumour progression, from a tumour suppressor in less advanced tumours to a promoter in more aggressive cancers 18. The paradoxical roles of TGF‐β are highlighted in the results presented in our research. It is well known that TGF‐β is a powerful immunosuppressor, which suppresses the activities of immune cells to provide a immune privilege microenvironment for cancer cells 28. Our previous works reported that TGF‐β down‐regulated the expression of HLA‐I and thereby attenuated the cytotoxic T cell‐mediated lysis of prostate cancer cells 29. Other studies also reported that TGF‐β decreased the expression of NKG2D and its ligands in some different tumour types 20, 30, 31. Together, TGF‐β might have different effects on NKG2DL transcription, and thus, different effects on the expression of NKG2DLs were determined. In the early stage of tumour development, TGF‐β up‐regulated the expression of SP1 and further promoted the transcription of NKG2DLs. With the development and metastases of tumour, the positive effect of TGF‐β can be reversed and the immunosuppressive effect of TGF‐β would be enhanced.

SP1 is a key transcription factor for MICA/B and ULBP1 and also a crucial positive regulator of NKG2DL in cells underwent EMT 14, 21. SP1 binds to GC promoter elements through three C(2)H(2)‐type zinc fingers presented at their C‐terminal domains. In this study, we detected the protein and mRNA levels of SP1, which is the crucial regulator of NKG2DL up‐regulation in cells underwent EMT. The protein, while not mRNA level of SP1 exhibited a rapid response to TGF‐β stimulation. Accordingly, silencing of SP1 by siRNA attenuated TGF‐β‐induced up‐regulation of NKG2DLs. These results suggested that there is a functional linkage between SP1 expression and TGF‐β‐induced up‐regulation of NKG2DLs in PC3 and HepG2 cells. SP1 plays a critical role in growth and metastasis of many tumours by modulating the expression of cell cycle genes and vascular endothelial growth factor 32. In addition, elevated SP1 expression was detected in tumours, whereas low SP1 expression was detected in stromal cells and normal glandular cells surrounding the tumours 33. SP1 is considered as a carcinogenic regulator and a potential target for cancer chemotherapy 34. Our present work revealed that overexpression of SP1 not only has the effect on malignant tumour, but also plays an important role in regulation of NKG2DLs expressed on tumour cells.

SP1 is a highly unstable protein with a short half‐life and regulated by a complex signalling network at both transcriptional and post‐transcriptional levels 35, 36. It is suggested that TGF‐β has feedback effects with SP1. SP1 is able to directly control the expression of TGF‐β and its receptor. On the other hand, TGF‐β enhances SP1 binding to GC boxes of the target genes 37, 38, 39. In this study, we demonstrated that TGF‐β stabilized SP1 *via* post‐transcriptional regulation process. SUMOylation and ubiquitination are the post‐translational modifications involved in SP1 cellular processes such as nuclear‐cytosolic transport, protein stability and transcriptional regulation 40, 41. We found that TGF‐β suppressed the ubiquitylation of SP1. As SP1 increases ubiquitination by altering its subcellular localization, we found that TGF‐β significantly increased the nuclear translocation of SP1. Together, TGF‐β promoted SP1 binding to GC boxes of NKG2DLs and then up‐regulated the expression of NKG2DLs in cancer cells.

**Figure 6:**
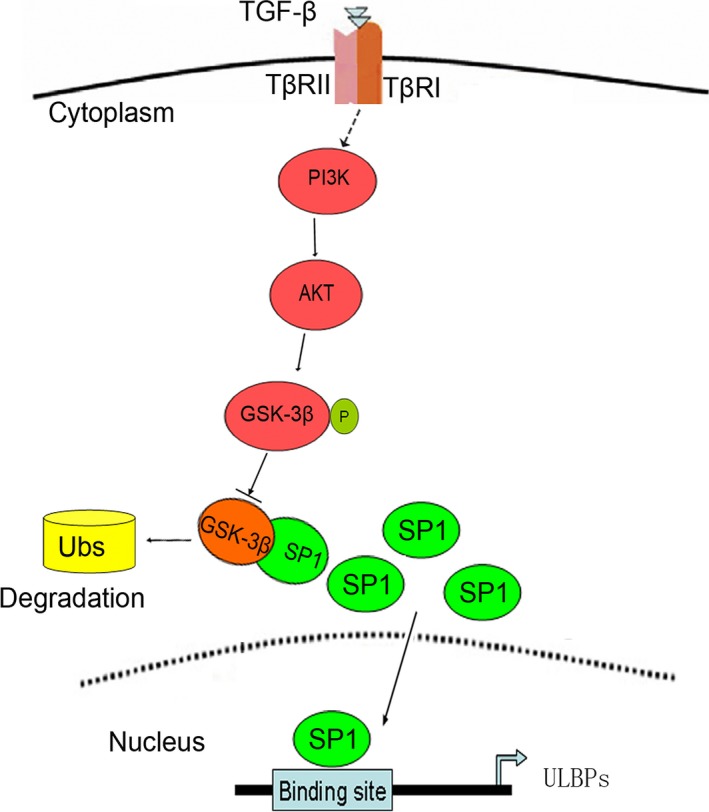
Model depicting the contribution of transforming growth factor beta (TGF‐β) to SP1‐mediated up‐regulation of NKG2DL transcription in the present study.

We further investigated the molecular mechanisms underlying TGF‐β mediated SP1 stabilization. The TGF‐β‐enhanced SP1 was blocked by treatment with TGF‐β type I receptor inhibitor or PI3K inhibitor, showing that TGF‐β type I receptor and PI3K/AKT signalling pathways are involved in TGF‐β‐stabilized SP1 in PC3 and HepG2 cells. These results suggested TGF‐β directly activated its receptor and thereby further activated PI3K/AKT signalling pathway. GSK‐3β is the main downstream kinase of AKT 42. In addition, it was found that inhibition of GSK‐3 increases NKG2D ligand MICA expression in multiple myeloma cells *via* a down‐regulation of STAT3 activation 43. In our present work, we found that activation of upstream AKT signalling represses GSK‐3β activity. Immunoprecipitation results confirmed that TGF‐β inhibits the association of SP1 with GSK‐3β and thereby increases the stability of SP1 as summarized in Fig. 6. After knockdown of GSK‐3β, TGF‐β‐mediated SP1 stabilization is not further elevated. In combination with the published literatures that SP1 and GSK‐3β have some indirect interactions 44, 45, our study suggested that GSK‐3β is involved in TGF‐β induced up‐regulation of NKG2DLs and stabilization of SP1 *via* direct or indirect manners.

In summary, we demonstrated that SP1 plays a critical role in TGF‐β‐induced up‐regulation of NKG2DLs in PC3 and HepG2 cells. We further showed that TGF‐β up‐regulates SP1 by increasing its stability in two ways. On the one hand, TGF‐β directly binds to its receptor, activates PI3K/AKT signalling pathway and subsequently represses GSK‐3β activity. On the other hand, TGF‐β inhibits the association of SP1 with GSK‐3β. These discoveries show the underlying mechanisms in TGF‐β‐induced up‐regulation of NKG2DLs in PC3 and HepG2 cells, which provides valuable evidence for exploring the tumour immune modulation function of TGF‐β.

## Conflict of interest

All the authors declare that there is no conflict of interest.
